# Three New Iridoid Derivatives Have Been Isolated from the Stems of *Neonauclea reticulata* (Havil.) Merr. with Cytotoxic Activity on Hepatocellular Carcinoma Cells

**DOI:** 10.3390/molecules23092297

**Published:** 2018-09-08

**Authors:** Fang-Pin Chang, Wei Chao, Sheng-Yang Wang, Hui-Chi Huang, Ping-Jyun Sung, Jih-Jung Chen, Ming-Jen Cheng, Guan-Jhong Huang, Yueh-Hsiung Kuo

**Affiliations:** 1 The Ph.D Program for Cancer Biology and Drug Discovery, China Medical University and Academia Sinica, Taichung 404, Taiwan; u101049002@cmu.edu.tw; 2 Graduate Institute of Medical Sciences, College of Medicine, Taipei Medical University, Taipei 250, Taiwan; sebrina0427@hotmail.com; 3 Department of Forestry, National Chung Hsing University, Taichung 402, Taiwan; taiwanfir@dragon.nchu.edu.tw; 4 Agricultural Biotechnology Research Center, Academia Sinica, Taipei 115, Taiwan; 5 Department of Chinese Pharmaceutical Sciences and Chinese Medicine Resources, China Medical University, Taichung 404, Taiwan; hchuang@mail.cmu.edu.tw; 6 National Museum of Marine Biology and Aquarium, Pingtung 912, Taiwan; pjsung@nmmba.gov.tw; 7 Graduate Institute of Marine Biology, National Dong Hwa University, Hualien 97401, Taiwan; 8 Faculty of Pharmacy, School of Pharmaceutical Sciences, National Yang-Ming University, Taipei 112, Taiwan; chenjj@ym.edu.tw; 9 Bioresource Collection and Research Center (BCRC), Food Industry Research and Development Institute (FIRDI), Hsinchu 300, Taiwan; chengmingjen2001@yahoo.com.tw; 10 Department of Biotechnology, Asia University, Taichung 413, Taiwan; 11 Chinese Medicine Research Center, China Medical University, Taichung 404, Taiwan; 12 Research Center for Chinese Herbal Medicine, China Medical University, Taichung 404, Taiwan

**Keywords:** *Neonauclea reticulate*, iridoid, neonanin, Hep3B

## Abstract

Three new iridoids, namely neonanin A (**1**), neonanin B (**2**) and neoretinin A (**3**), as well as twelve known compounds, 6-hydroxy-7-methyl-1-oxo-4-carbomethoxyoctahydrocyclopenta[*c*]pyran (**4**), 4-*epi*-alyxialactone (**5**), loganetin (**6**), loganin (**7**), phenylcoumaran-α′-aldehyde (**8**), cleomiscosin A (**9**), ficusal (**10**), balanophonin (**11**), vanillic acid (**12**), *p*-coumaric acid (**13**), *cis,trans*-abscisic acid (**14**), and *trans,trans*-abscisic acid (**15**) were isolated from the stems of *Neonauclea reticulata* (Havil.) Merr. These new structures were determined by the detailed analysis of spectroscopic data and comparison with the data of known analogues. Compounds 1–13 were evaluated using an in-vitro MTT cytotoxic assay for hepatocellular carcinoma (HCC) cells, and the preliminary results showed that ficusal (10), balanophonin (11), and *p*-coumaric acid (13) exhibited moderate cytotoxic activity, with EC_50_ values of 85.36 ± 4.36, 92.63 ± 1.41, and 29.18 ± 3.48 µg/mL against Hep3B cells, respectively.

## 1. Introduction

*Neonauclea reticulate* (Havil.) Merr. is a large evergreen tree, which is distributed over the Philippines and Taiwan. Of the forty species of the genus *Neonauclea* (Rubiaceae), this is the only species that can be found in Taiwan, located in the forests at low elevations of southern Taiwan, such as the Kaohsiung area, as well as Pingtung Mountain or Orchid Island [1]. On Orchid Island, when the Tao people celebrate the flying fish festival, this tree is an important folk plant for building the Tribe’s *chinurikuran*. Alkaloids [2,3], anthraquinones [4], iridoids [5], triterpenes [6], and saponins [7] have been isolated from the plants of this genus in previous chemical investigations. Anti-bacterial [8], anti-malarial [9], and anti-topoisomerase II effects [4] were shown in previous pharmacological studies. To the best of our knowledge, there have been no studies published that have investigated the chemical structure of *N. reticulate*. Furthermore, the only pharmacological study published mentions that the leaves of this plant could protect human skin fibroblast cells against the effects of ultraviolet B (UVB) irradiation [10]. Further investigations of the chemical and pharmacological properties of *N. reticulate* are urgently needed.

A global estimation report has proposed that hepatocellular carcinoma (HCC) will be one of the leading causes of cancer-associated deaths in 2018 [11]. In Taiwan, HCC is the second most common cancer in males and fourth most common cancer in females, according to the cancer registry annual report for the year 2015 [12]. HepG2 and Hep3B cell lines are the well-known in vitro cytotoxicity assay models, and they are the most well-characterized liver cancer cell lines. These two cell lines are very similar, except that HepG2 is hepatitis B virus-negative and non-tumorigenic, while Hep3B is hepatitis B virus-positive and tumorigenic. These two cell lines showed different chemo-sensitivity in cytotoxicity, gene expression induction, and cell cycle response and biochemical effects, which may provide investigators further instruction for identifying the mechanism [13]. However, there are only a few medication choices for HCC, including molecularly targeted therapy, such as sorafenib; immunotherapy, such as nivolumab; and cytotoxic chemotherapy, such as doxorubicin [14]. Finding possible compounds for HCC treatment demands immediate attention.

In previous studies, natural resources for the treatment of hepatocellular carcinoma have been found in the members of Rubiaceae family, such as *Oldenlandia diffusa* [15] and *Paederia scandens* [16]. The phytochemical studies of *N. reticulate* have not yet been performed. Thus, the aim of this study is to investigate the compounds from the stems of *N. reticulate* and its preliminary in-vitro cytotoxicity analysis against hepatocellular carcinoma cells.

## 2. Results and Discussions

### 2.1. Isolation and Structural Elucidation

**Figure 1:**
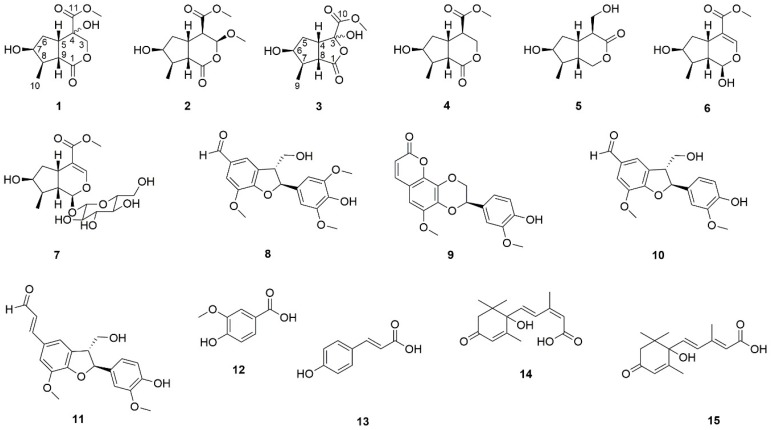
The chemical structures of compounds **1**–**15** from *Neonauclea reticulata* (Havil.) Merr.

In this study, we used the human hepatocellular carcinoma cell lines of HepG2 and Hep3B to evaluate the cytotoxicity of the MeOH extract. The results show that the MeOH extract exhibits a dose-dependent cytotoxicity against the Hep3B cells (effective concentration dose; EC_50_ = 912.98 ± 3.95 μg/mL). In contrast, there was no significant cytotoxicity in Hep G2 cells. These biological assays suggest that the extract of *N. reticulate* might inhibit the growth of the Hep3B cell line. Therefore, the extract was suspended in water and partitioned with ethyl acetate (EtOAc). After being suspended in water, this extract was successively partitioned with butanol, with each step being repeated three times. Three different fractions were used to evaluate the cytotoxicity in Hep3B cells. Among them, only the EtOAc fraction revealed a significant inhibitory effect (EC_50_ = 591.13 ± 4.99 μg/mL). The other fractions (BuOH and H_2_O) showed no significant cytotoxicity in Hep3B cells. The investigation of the active fraction was isolated by silica gel column chromatography and normal-phase, semi-preparative, high-performance liquid chromatography (HPLC) to obtain fifteen compounds. Among these compounds, we isolated three new iridoids, which are namely neonanin A (**1**), neonanin B (**2**) and neoretinin A (**3**), and twelve known compounds, including 6-hydroxy-7-methyl-1-oxo-4-carbomethoxyoctahydrocyclopenta[c]pyran (**4**) 4-*epi*-alyxialactone (**5**), loganetin, loganin (**7**), phenylcoumaran-α′-aldehyde (**8**), cleomiscosin A (**9**), ficusal (**10**), balanophonin (**11**), vanillic acid (**12**), *p*-coumaric acid (**13**), *cis*,*trans*-abscisic acid (**14**), and *trans*,*trans*-abscisic acid (**15**). The structures of the new compounds were determined through spectral analyses, including IR, UV, one-dimensional (1D) and two-dimensional (2D)-NMR, as well as HR-ESI-MS data. The known compounds identified were compared with the published NMR spectral data. All structures are shown in Figure 1. In this study, we described the detailed structural elucidations of new compounds and the activities of compounds **1**–**13**.

Compound **1** was obtained as a colorless oil. The molecular weight was determined by HR-ESI-MS, which showed an [M + Na]^+^ ion at an *m*/*z* of 267.0845 (calculated for C_11_H_16_O_6_Na 267.0839), indicating four degrees of unsaturation. The IR spectrum displayed the presence of hydroxyl (3491 cm^−1^) and carbonyl (1728 cm^−1^) functionalities. The ^1^H spectra of compound **1** (Table 1) showed a doublet methyl group δ_H_ 1.23 (d, *J* = 7.1, H-10); one pair of geminal coupling methylene groups at δ_H_ 1.53 (m, H-6α) and δ_H_ 2.01 (m, H-6β); two methine protons of δ_H_ 2.88 (dd, *J* = 9.9, 7.1, H-9) and δ_H_ 3.31 (ddd, *J* = 9.9, 8.0, H-5); a singlet carbomethoxy group at δ_H_ 3.81 (s, COOMe); one pair of mutual geminally coupled oxymethylene signals at δ_H_ 3.87 (d, *J* = 12.0, H-3α) and δ_H_ 3.97 (d, *J* = 12.0, H-3β); and an oxygenated methine proton δ_H_ 4.22 (m, H-7). Eleven carbons were assessed using ^13^C-NMR (Table 1), and the heteronuclear single quantum coherence spectroscopy (HSQC) spectra revealed the presence of methyls δ_C_ 14.1 (C-10) and δ_C_ 52.8 (OMe); methylenes δ_C_ 37.8 (C-6) and δ_C_ 67.5 (C-3); methines δ_C_ 43.9 (C-5), δ_C_ 44.4 (C-8), and δ_C_ 50.6 (C-9); oxygenated methine δ_C_ 76.9 (C-7); oxygenated quaternary carbon δ_C_ 88.4 (C-4); and carbonyl groups δ_C_ 169.1 (C-11) and δ_C_ 178.5 (C-1). The correlation spectroscopy (COSY) correlations between H-5 (δ_H_ 3.31)/H-6 (δ_H_ 1.53 and δ_H_ 2.01) and H-9 (δ_H_ 2.88), H-6 (δ_H_ 1.53 and δ_H_ 2.01)/H-7 (δ_H_ 4.22), H-7 (δ_H_ 4.22)/H-8 (δ_H_ 2.29), H-8 (δ_H_ 2.29)/H-9 (δ_H_ 2.88), and H-10 (δ_H_ 1.23) suggests that there was a cyclopentanyl moiety with a methyl group and a hydroxyl group in the structure. We found that there were heteronuclear multiple bond coherence (HMBC) correlations between H-3 (δ_H_ 3.87 and δ_H_ 3.97)/C-4 (δ_C_ 88.4) and C-11 (δ_C_ 169.1); H-5 (δ_C_ 3.31)/C-1 (δ_C_ 178.5), C-3 (δ_C_ 67.5), and C-6 (δ_C_ 37.8); H-9 (δ_C_ 2.88)/C-1 (δ_C_ 169.1), C-6 (δ_C_ 37.8), and C-10 (δ_C_ 14.1); and OMe (δ_H_ 3.81)/C-11 (δ_C_ 169.1). These correlations indicated that there was another pyranyl moiety with a lactone and a carbomethoxy group, which included the COSY and HMBC information. This indicates that a cyclopentan and a pyranyl moiety were connected to each other in compound **1.** This spectra information for compound **1** was very similar to those of the compound **4** [17]. Thus, we suggested that compound **1** has an iridoid type structure. The difference between these two compounds was in the C-4 position. The C-4 position of compound **4** was a tertiary carbon (δ_C_ 46.1), while in compound **1** a quaternary carbon with an oxygen atom (δ_C_ 88.4) were connected in the C-4 position. It is difficult to elucidate the relative configuration of C-4 in compound **1** using the present spectra data. According to the nuclear overhauser effect spectroscopy (NOESY) spectra, H-6α/H-7/H-8 were in the α-configuration, while H-5, H-6β, and H_3_-10 were all in the β form. By consolidating the above-mentioned results and comparing them to the literature, compound **1** was assigned to be neonanin A.

**Figure 2:**
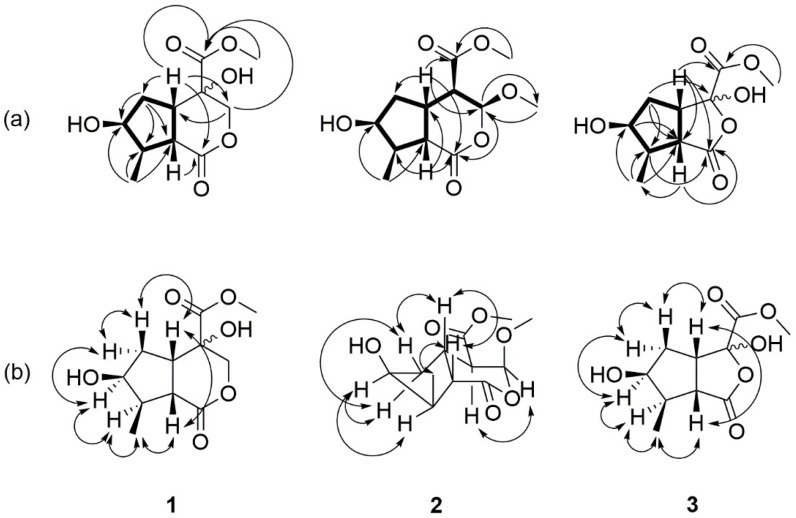
(**a**) Significant correlation spectroscopy (COSY) (bold line) and heteronuclear multiple bond coherence (HMBC) (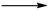) correlations for compounds **1**–**3** (**b**). Significant NOESY (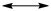) correlations of compounds **1**–**3**.

Compound **2** was obtained as a white needle with an MP of approximately 82–84 °C. The molecular weight was determined by HR-ESI-MS, which showed an [M + Na]^+^ ion at an *m/z* of 281.0999 (calculated for C_12_H_18_O_6_Na 281.0996), indicating four degrees of unsaturation. The IR spectrum displayed the presence of hydroxyl (3392 cm^−1^) and ester carbonyl (1730 cm^−1^) functionalities. The ^1^H and ^13^C-NMR spectra of compound **2** (Table 1) showed that there is a doublet methyl group δ_H_ 1.28 (d, *J* = 6.9, H-10); one pair of geminal coupled methylene groups—δ_H_ 1.38 (m, H-6α) and δ_H_ 2.34 (m, H-6β); two methine protons, δ_H_ 2.79 (dd, *J* = 11.4, 9.1, H-9) and δ_H_ 3.28 (m, H-5); two methoxy groups, δ_H_ 3.51 (s, OMe) and δ_H_ 3.76 (s, COOMe); an oxygenated methine proton δ_H_ 4.12 (m, H-7); and an acetal proton δ_H_ 5.42 (d, *J* = 2.4, H-3). There were twelve carbons found in ^13^C-NMR after being combined with distortionless enhancement by polarization transfer (DEPT). There were two ester carbonyl groups: δ_C_ 173.9 (C-1) and δ_C_ 169.8 (C-11). The COSY spectra found correlations between H-5 (δ_H_ 3.28)/H-6 (δ_H_ 1.38 and δ_H_ 2.34) and H-9 (δ_H_ 2.79); H-6 (δ_H_ 1.38 and δ_H_ 2.34)/H-7 (δ_H_ 4.12); H-7 (δ_H_ 4.12)/H-8 (δ_H_ 2.27); and H-8 (δ_H_ 2.27)/H-9 (δ_H_ 2.79) and H-10 (δ_H_ 1.28). The HMBC spectrum found correlations between H-3 (δ_H_ 5.42)/C-1 (δ_C_ 173.9), C-4 (δ_C_ 49.8), and C-11 (δ_C_ 169.8); H-5 (δ_H_ 2.79)/C-1 (δ_C_ 173.9), C-3 (δ_C_ 101.9), and C-6 (δ_C_ 42.1); and H-9 (δ_H_ 2.79)/C-1 (δ_C_ 173.9), C-6 (δ_C_ 42.1), and C-10 (δ_C_ 1.28). The correlations found in HMBC spectra indicate the presence of a methoxy group (δ_H_ 3.51) in the C-3 position. These spectra and corrections were very similar to compound **4**, which indicates that compound **2** is also an iridoid type compound, except for the addition of a methoxy group located in the C-3 position. H-4 exhibited the two coupling constants, with *J* = 11.4 at 2.4 Hz. The coupling constant of H-4 showed a coupling constant of *J* = 2.4 Hz. Thus, the coupling constant with *J* = 11.4 Hz represents the coupling between H-4 and H-5. The evidence concluded that H-4 is located in the α-axial configuration. The results provided support for H-3 being in the α-equatorial orientation. The NOESY spectrum (Figure 2) provides further evidence for two protons, which was presented by two α-configuration. The compound **2** was assigned to be neonanin B.

**Figure 3:**
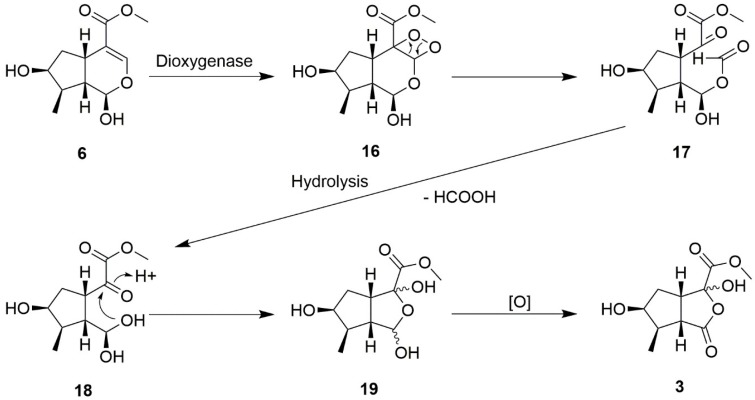
Proposed biosynthetic sequence of neoretinin A (**3**).

Compound **3** was obtained as a colorless oil. The molecular weight was determined by HR-ESI-MS, which showed an [M + Na]^+^ ion at *m*/*z* of 253.0685 (calculated for C_10_H_14_O_6_Na 253.0683), indicating four degrees of unsaturation. The IR spectrum displays the presence of hydroxyl (3361 cm^−1^) and lactone carbonyl (1741 cm^−1^) functionalities. The ^1^H and ^13^C-NMR spectra of compound **3** (Table 1) shows that there is a doublet methyl group δ_H_ 1.23 (d, *J* = 7.0, H-9); two methylene protons δ_H_ 2.25 (m, H-5α) and δ_H_ 1.88 (m, H-5β); one carbomethoxy group δ_H_ 3.88 (s, COOMe); a methine proton δ_H_ 2.99 (t, *J* = 8.5, H-8); and a hydroxyl group δ_H_ 4.92 (brs). Ten carbons were shown in ^13^C-NMR after being combined with DEPT. There was a quaternary carbon δ_C_ 100.3 (C-3) and two carbonyl groups δ_C_ 176.7 (C-1) and δ_C_ 170.1 (C-10). The COSY spectra showed correlations between H-4 (δ_H_ 3.47)/H-5 (δ_H_ 1.88 and 2.25) and H-8 (δ_H_ 2.99); H-5 (δ_H_ 1.88 and 2.25)/H-6 (δ_H_ 4.29); H-6 (δ_H_ 4.29)/H-7 (δ_H_ 2.36); and H-7 (δ_H_ 2.36)/H-8 (δ_H_ 2.99) and H-9 (δ_H_ 1.23). This indicates that there was also a cyclopentane ring in the structure of compound **3**. The key HMBC spectra correlations were between H-4 (δ_H_ 3.47)/C-1 (δ_C_ 176.7), C-3 (δ_C_ 100.3), and C-10 (δ_C_ 170.1); H-5α(δ_H_ 2.25)/C-3 (δ_C_ 100.3); and H-8 (δ_H_ 2.99)/C-1 (δ_C_ 176.7). A possible biosynthetic pathway of compound **3** was proposed as illustrated in Figure 3. Loganetin (**6**) was oxidized by a dioxygenase enzyme to yield dioxetane (compound **16**), before being spontaneously cleaved to yield the intermediate compound **17**. After hydrolysis, the formulate was abandoned and formed compound **18**, which condensed to obtain diacetal (compound **19**). Subsequently, compound **19** was partially oxidized to obtain compound **3**. From the literature survey, no report has mentioned this type of structure, so we suggested that this compound **3** was a new skeleton and named it 3-nor(2→4)abeoiridoid. The compound **3** was assigned as neoretinin A.

### 2.2. Structural Identification of Known Isolates

The known isolates were readily identified by the comparison of their spectroscopic data with those of the corresponding authentic samples or literatures. They include the following twelve compounds: 6-hydroxy-7-methyl-1-oxo-4-carbomethoxyoctahydrocyclopenta[c]pyran (**4**) [17], 4-*epi*-alyxialactone (**5**) [18], loganetin (**6**) [19], loganin (**7**) [20], phenylcoumaran-α′-aldehyde (**8**) [21], cleomiscosin A (**9**) [22], ficusal (**10**) [23], balanophonin (**11**) [24], vanillic acid (**12**) [25], *p*-coumaric acid (**13**) [26], *cis,trans*-abscisic acid (**14**), and *trans,trans*-abscisic acid (**15**) [27].

### 2.3. In-Vitro Cytotoxic Activity Against Hep3B Cells

In this study, the cytotoxic abilities of seven iridoids (**1**–**7**), four neolignanes (**8**–**11**), and two aromatic rings (**12**–**13**) against Hep 3B cells were shown in Table 2. The three compounds, which are namely ficusal (**10**), balanophonin (**11**), and p-coumaric acid (**13**), exhibited moderate inhibitory effects, with EC_50_ values of 85.36 ± 4.36, 92.63 ± 1.41, and 29.18 ± 3.48 µg/mL, respectively. Doxorubicine was used as a positive control, which is an anthracycline antibiotic used for hepatocellular carcinoma therapy. The main mechanism is intercalant transcription, which inhibits the effectivity and inhibition of topoisomerase II activity [28]. The EC_50_ value of doxorubicine was 0.31 ± 0.08 µg/mL. Only one study published recently mentioned that balanophonin (**10**) has cytotoxic ability with regard to a Hep3B cell with IC_50_ = 29.3 ± 0.2 µM (equal to 10.44 ± 0.2 µg/mL). These inconsistent findings might be related to different study designs, laboratory performing skills, and experimental environment [29].

## 3. Materials and Methods

### 3.1. General

The mass spectrometric (HR-ESI-MS) data were generated at the Mass Spectrometry Laboratory of the Chung Hsing University with a Thermo LTQ Orbitrap XL™ Hybrid Ion Trap-Orbitrap Mass Spectrometer (Thermo Scientific Inc., Waltham, MA, USA). The melting point data were obtained with the melting point apparatus MP-S3 (YANACO Inc, Kyoto, Japan). The specific rotation data were obtained with a Jasco P-2000 Polarimeter (JASCO Inc., Tokyo, Japan). The infrared spectra were obtained with a Shimadzu IRAffinity-1S Fourier Transform Infrared Spectrophotometer (Shimadzu Inc., Kyoto, Japan). The UV spectra were obtained with a Shimadzu 160A UV–Visible recording spectrophotometer. The 1D and 2D-NMR spectra were recorded with a Bruker Avance 500 FT-NMR spectrometer (Bruker Inc., Bremen, Germany). Column chromatography was performed using LiChroCART Si 5 µM gel (Merck, Darmstadt, Germany) and Sephadex LH-20 (GE Healthcare Life Sciences Inc., Marlborough, MA, USA). The TLC (thin-layer chromatography) analysis was carried out using aluminum pre-coated Si plates (Silica Gel 60 F-254; Merck). The spots were visualized using a UV lamp at λ = 254 nm and detected by spraying with 10% H_2_SO_4_ alcohol solution, before heating at 125 °C. Semi-preparative HPLC was performed using a normal phase column (Luna 5μm Silica 100 Å, 250 × 10 mm; Phenomenex Inc., Torrance, CA, USA) on a Precision Instruments IOTA 2 Refractive Index Detector system.

### 3.2. Chemicals

The solvents used to open the column isolation (Silica gel and Sephadex LH 20 gel column) in the study, such as *n*-hexane, chloroform, ethyl acetate, acetone, and methanol, were of ACS grade. The *n*-hexane, chloroform, and acetone used for HPLC isolation, which was of HPLC grade, and the deuterated solvents for NMR measurement (CDCl_3_ and CD_3_COCD_3_) were purchased from the branch of Merck in Taipei, MTT (3-(4,5-dimethylthiazol-2-yl)-2,5-diphenyltetrazolium bromide). Doxorubicin and other chemicals were purchased from Sigma Chemical Co. (St. Louis, MO, USA). Minimum essential media (MEM), trypsin–EDTA (ethylenediaminetetraacetic acid), fetal bovine serum (FBS), penicillin/streptomycin, non–essential amino acids (NEAA), and sodium pyruvate were obtained from Gibco (BRL life Technologies, Grand Island, NY, USA).

### 3.3. Plant Material

The stems of Neonauclea reticulate were collected from Nan Ren Mountain, Pingtung, Taiwan, in August 2012, and identified by Yau Lun Kuo (Professor, Department of Forestry, National Pingtung University of Science and Technology, Pingtung, Taiwan). A voucher specimen (CMU-NR-201208) was deposited at the School of Chinese Pharmaceutical Sciences and Chinese Medicine Resources.

### 3.4. Extraction and Isolation

The dried stems of Neonauclea reticulate (9.0 kg) were extracted with MeOH (50 L each for seven days) three times. The MeOH extract was concentrated under reduced pressure at 35 °C, before the residue (365 g) was partitioned between EtOAc and H_2_O (1:1) to provide the EtOAc-soluble fraction (100 g). The water suspension was partitioned again with butanol (1:1) to get the BuOH-soluble fraction (152 g) and the H_2_O-soluble fraction (98 g). The EtOAc-soluble fraction was purified by column chromatography (CC) (2.0 kg of SiO_2_, 70–230 mesh; *n*-hexane/EtOAc/methanol gradient) to allow 32 fractions, Fr.1–Fr.32. Fr.22 (4.6 g) was re-separated by sephadex LH-20 (250 g; CHCl_3_/MeOH = 3/7) to produce 11 fractions of Fr.22-1–Fr.22-11. Fr.22-6 (1.47 g) was re-separatecd by silica gel column chromatography (30 g of SiO_2_, 70–230 mesh; CHCl_3_/EtOAc (25%)) and purified by normal-phase HPLC (*n*-hexane/acetone (35%)) to afford pure compounds **2** (9.5 mg, t_R_ = 11 min), **3** (1.7 mg, t_R_ = 16 min), and **4** (6.1 mg, t_R_ = 14 min). Fr.22-7 (275.0 mg) was re-separatecd by silica gel column chromatography (5.5 g of SiO_2_, 70–230 mesh; CHCl_3_/acetone (20%)) to afford fourteen fractions of Fr.22-7-1–Fr.22-7-14. Fr.22-7-9 affords pure compound **9** (8.5 mg, R_f_ = 0.2). Fr.22-7-8 (7.4 mg) was purified by normal phase HPLC (*n*-hexane/acetone (30%)) to form pure compounds **10** (3.2 mg, t_R_ = 15 min) and **11** (2.0 mg, t_R_ = 25 min). Fr.22-9 (141.2 mg) was purified by normal phase HPLC (CHCl_3_/acetone (20%)), resulting in pure compounds **12** (14.2 mg, t_R_ = 10 min) and **13** (2.8 mg, t_R_ = 9 min). Fr.23 (1.2 g) was re-separated by sephadex LH-20 (140 g; CHCl_3_/MeOH = 3/7) to afford the six fractions Fr.23-1–Fr.23-6. Fr.23-3 (832.68 mg) was re-separatecd by silica gel column chromatography (30 g of SiO_2_, 70–230 mesh; CHCl_3_/EtOAc (25%)) and purified by normal-phase HPLC (*n*-hexane/acetone (35%)), in order to afford pure compounds **1** (1.1 mg, t_R_ = 28 min), **5** (30.3 mg, t_R_ = 20 min), **6** (27.6 mg, t_R_ = 13 min), **8** (3.0 mg, t_R_ = 32 min), **14** (1.2 mg, t_R_ = 16 min), and **15** (0.8 mg, t_R_ = 11 min). Fr.29 (4.9 g) was washed with acetone and filtered to produce a white residue. The white residue was found to be pure compound **7** (0.5 g).

neonanin A (**1**): colorless oil; ${\left[\alpha \right]}_{D}^{28}$ + 17.6 (*c* 0.10, MeOH); IR (KBr) ν_max_: 3491, 2962, 1728, 1438, and 1060 cm^−1^; HR-ESI-MS *m*/*z* 267.0845 [M + Na]^+^ (calculated for C_11_H_16_O_6_Na at 267.0839); ^1^H-NMR and ^13^C-NMR (500/125 MHz, in CDCl_3_) are shown on Table 1.

neonanin B (**2**): white needle; MP: 82–84 °C; ${\left[\alpha \right]}_{D}^{27}$ + 93.6 (*c* 0.10, MeOH); IR (KBr) ν_max_: 3392, 1730, 1444, 1371, and 1172 cm^−1^; HR-ESI-MS *m*/*z* 281.0999 [M + Na]^+^ (calculated for C_12_H_18_O_6_Na at 281.0996); ^1^H-NMR and ^13^C-NMR (500/125 MHz, in CDCl_3_) are shown on Table 1.

neoretinin A (**3**): colorless oil; ${\left[\alpha \right]}_{D}^{27}$ − 73.1 (*c* 0.10, MeOH); IR (KBr) ν_max_: 3361, 2939, 1741, 1450, 1205, and 1024 cm^−1^; HR-ESI-MS *m*/*z* 253.0685 [M + Na]^+^ (calculated for C_10_H_14_O_6_Na at 253.0683); ^1^H-NMR and ^13^C-NMR (500/125 MHz, in CDCl_3_) are shown on Table 1.

### 3.5. Cell Culture

Hep3B (Bioresource Collection and Research Center (BCRC) Number: 60434) and HepG2 (BCRC Number: 60364) cells were obtained from Food Industry Research and Development Institute (Hsin Chu, Taiwan). All of the cell lines were cultured in MEM containing 10% (*v*:*v*) FBS, penicillin/streptomycin (100 U/mL), 0.1 mM NEAA, and 1.0 mM sodium pyruvate. The cells were cultured in a humidified incubator under 5% CO_2_ at 37 °C.

### 3.6. Cytotoxic Assay

The in-vitro cytotoxic activity of MeOH extracts, partition fractions, and pure compounds were determined by the MTT assay. Hep3B (2 × 10^4^/well) and HepG2 (1 × 10^4^/well) cells were seeded in 96-well plates and incubated for 24 h. Both cells were treated with MeOH extracts that contain partition fractions in various concentrations (0, 62.5, 125, 250, 500, and 1000 μg/mL). Furthermore, each pure compound was evaluated only in Hep3B cells, and the dosages used were 0, 6.25, 12.5, 25, 50, and 100 μg/mL. After 48 h, the medium was replaced with a medium containing 0.5 mg/mL MTT solution and incubated at 37 °C for 4 h. After the end of the MTT reaction, we used isopropanol/HCl solution to dissolve the formazan crystals. The absorbance was measured spectrophotometrically at 570 nm. The cytotoxicity was calculated and compared with the control group.

### 3.7. Statistical Analysis

The results were presented as mean values ± SD (standard deviations) of at least three independent experiments. Statistical analyses were performed using Microsoft Excel 2010 software (Information Center, China Medical University, Taichung, Taiwan).

## 4. Conclusions

The isolation and structural elucidation of fifteen compounds, including three new compounds—namely neonanin A (**1**)**,** neonanin B (**2**) and neoretinin A (**3**)—as well as twelve known compounds—6-hydroxy-7-methyl-1-oxo-4-carbomethoxyoctahydrocyclopenta[c]pyran (**4**), 4-*epi*-alyxialactone (**5**), loganetin (**6**), loganin (**7**), phenylcoumaran-α′-aldehyde (**8**), cleomiscosin A (**9**), ficusal (**10**), balanophonin (**11**), vanillic acid (**12**), *p*-coumaric acid (**13**), *cis,trans*-abscisic acid (**14**), and *trans,trans*-abscisic acid (**15**)—were isolated from the stems of *Neonauclea reticulata* (Havil.) Merr. The structures of these compounds were established based on the spectroscopic data. Three of the compounds, namely ficusal (**10**), balanophonin (**11**), and *p*-coumaric acid (**13**) exhibited moderate cytotoxicity, with EC_50_ values of 85.36 ± 4.36, 92.63 ± 1.41, and 29.18 ± 3.48 µg/mL, respectively, against Hep3B cells within 48 h. To the best of our knowledge, this is the first study that has conducted phytochemical investigation of *Neonauclea reticulata* (Havil.) Merr. and provided preliminary results of cytotoxicity on hepatocellular carcinoma cells.

## Figures and Tables

**Table 1:** NMR data (CDCl_3_) of compound **1**–**3** in ppm, with *J* in Hz.

	Compound 1		Compound 2			Compound 3	
Position	δ_H_	δ_C_	δ_H_	δ_C_	Position	δ_H_	δ_C_
1		178.5		173.9	1		176.7
2					2		
3α	3.87 (d, *J* = 12.0)	67.5	5.42 (d, *J* = 2.4)	101.9	3		100.3
3β	3.97 (d, *J* = 12.0)				4	3.47 (q, *J* = 8.5)	44.8
4		88.4	2.55 (dd, *J* = 11.4, 2.4)	49.8	5α	2.25 (m)	34.6
5	3.31 (ddd, *J* = 9.9, 8.0)	43.9	3.28 (m)	31.9	5β	1.88 (m)	
6α	1.53 (m)	37.8	1.38 (m)	42.1	6	4.29 (m)	76.0
6β	2.01 (m)		2.34 (m)		7	2.36 (m)	44.3
7	4.22 (m)	76.9	4.12 (m)	74.8	8	2.99 (t, *J* = 8.5)	51.6
8	2.29 (m)	44.4	2.27 (m)	43.1	9	1.23 (d, *J* = 7.0)	13.6
9	2.88 (dd, *J* = 9.9, 7.1)	50.6	2.79 (dd, *J* = 11.4, 9.1)	46.0	10		170.1
10	1.23 (d, *J* = 7.1)	14.1	1.28 (d, *J* = 6.9)	14.3	COO*Me*	3.88 (s)	54.1
11		169.1		169.8			
COO*Me*	3.81 (s)	52.8	3.51 (s)	57.1			
3-O*Me*			3.76 (s)	52.4			

**Table 2:** Effects of compounds isolated from *Neonauclea reticulate* and the cytotoxicity viability of Hep3B cell.

Compounds	EC_50_ (µg/mL) in 48 h
neonanin A (**1**)	>100
neonanin B (**2**)	>100
neoretinin A (**3**)	>100
6-hydroxy-7-methyl-1-oxo-4-carbomethoxyoctahydrocyclopenta[c]pyran (**4**)	>100
4-*epi*-alyxialactone (**5**)	>100
loganetin (**6**)	>100
loganin (**7**)	>100
phenylcoumaran-α′-aldehyde (**8**)	>100
cleomiscosin A (**9**)	>100
ficusal (**10**)	85.36 ± 4.36
balanophonin (**11**)	92.63 ± 1.41
vanillic acid (**12**)	>100
*p*-coumaric acid (**13**)	29.18 ± 3.48
Doxorubicin	0.31 ± 0.08

Values are expressed as mean ± SD of three replicates.

## Supplementary Materials

The following are available online: 1D- and 2D-NMR, as well as HR-ESI-MS spectra of Compounds **1**–**3**.
