# Supplementary material for: Three New Iridoid Derivatives Have Been Isolated from the Stems of Neonauclea reticulata (Havil.) Merr. with Cytotoxic Activity on Hepatocellular Carcinoma Cells
Source: Molecules. 2018 Sep 8;23(9):2297. doi: 10.3390/molecules23092297 (PMC6225429; doi:10.3390/molecules23092297)
Supplement: Supplementary file 1 [file molecules-23-02297-s001.pdf]

Supplementary Materials

Three New Iridoid Derivatives Have Been Isolated from the Stems of *Neonauclea reticulata* (Havil.) Merr. with Cytotoxic Activity on Hepatocellular Carcinoma Cells

Fang-Pin Chang [1], Wei Chao [2], Sheng-Yang Wang [3,4], Hui-Chi Huang [5], Ping-Jyun Sung [6,7], Jih-Jung Chen [8,†], Ming-Jen Cheng [9,†], Guan-Jhong Huang [5,*] and Yueh-Hsiung Kuo [1, 5, 10,11,12,*]

[1] The Ph.D Program for Cancer Biology and Drug Discovery, China Medical University and Academia Sinica, Taichung 404, Taiwan; u101049002@cmu.edu.tw

[2] Graduate Institute of Medical Sciences, College of Medicine, Taipei Medical University, Taipei 250, Taiwan; sebrina0427@hotmail.com

[3] Department of Forestry, National Chung Hsing University, Taichung 402, Taiwan ; taiwanfir@dragon.nchu.edu.tw

[4] Agricultural Biotechnology Research Center, Academia Sinica, Taipei 115, Taiwan

[5] Department of Chinese Pharmaceutical Sciences and Chinese Medicine Resources, China Medical University, Taichung 404, Taiwan; hchuang@mail.cmu.edu.tw (H.-C.H.); gjhuang@mail.cmu.edu.tw (G.-J.H.); kuoyh@mail.cmu.edu.tw(Y.-H.K.)

[6] National Museum of Marine Biology and Aquarium, Pingtung 912, Taiwan; pjsung@nmmba.gov.tw

[7] Graduate Institute of Marine Biology,National Dong Hwa University, Hualien 97401, Taiwan

[8] Faculty of Pharmacy, School of Pharmaceutical Sciences, National Yang-Ming University, Taipei, 112, Taiwan; chenjj@ym.edu.tw

[9] Bioresource Collection and Research Center (BCRC), Food Industry Research and Development Institute (FIRDI), Hsinchu 300, Taiwan; chengmingjen2001@yahoo.com.tw

[10] Department of Biotechnology, Asia University, Taichung 413, Taiwan

[11] Chinese Medicine Research Center, China Medical University, Taichung 404, Taiwan

[12] Research Center for Chinese Herbal Medicine, China Medical University, Taichung 404, Taiwan

* Correspondence: gjhuang@mail.cmu.edu.tw; Tel.: +886-4-2205-3366 (ext. 5508) (G.-J.H.); kuoyh@mail.cmu.edu.tw; Tel.: +886-4-2205-3366 (ext. 5701); Fax: +886-4-2207-1693 (Y.-H.K.)

† These authors contributed equally to this work

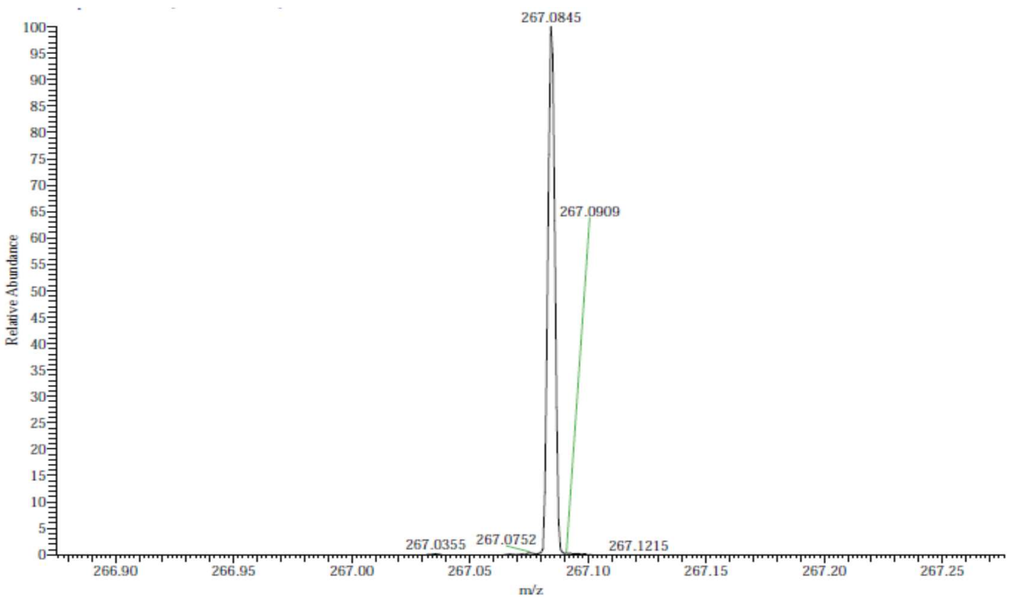

Fig. S1. HR-ESI-MS spectrum of compound **1**.

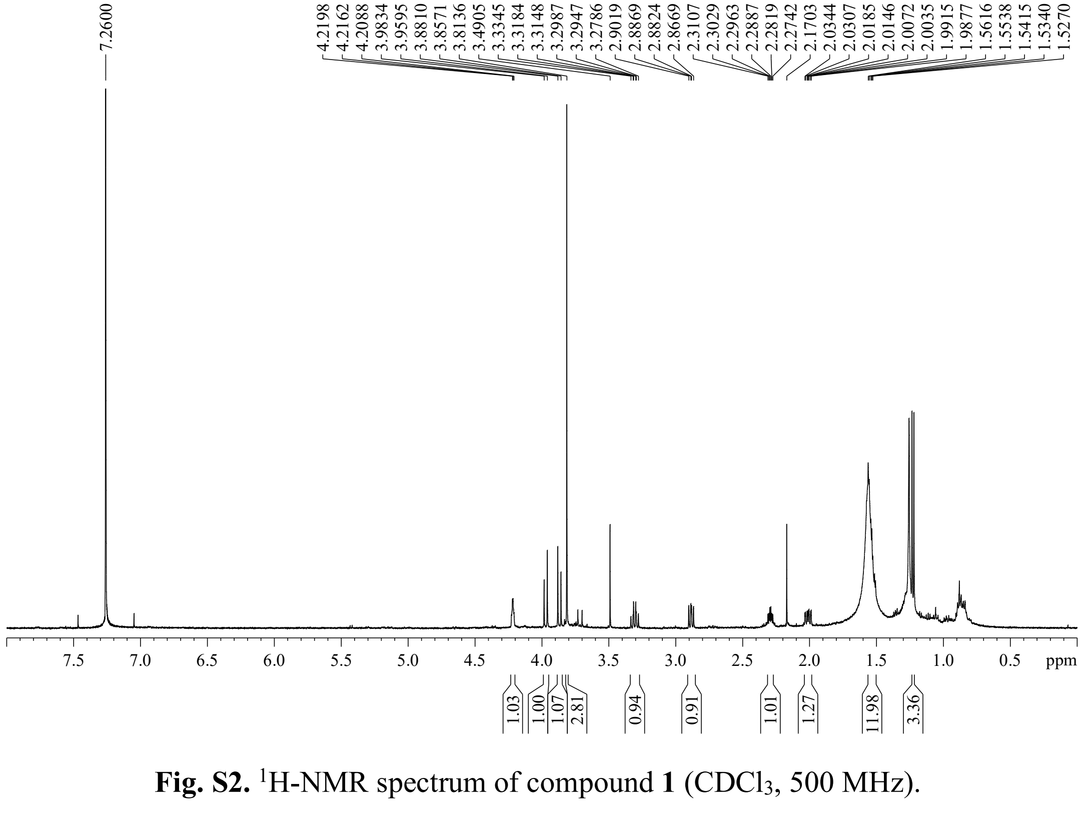

Fig. S2. ^{1}H-NMR spectrum of compound **1** ($CDCl_3$, 500 MHz).

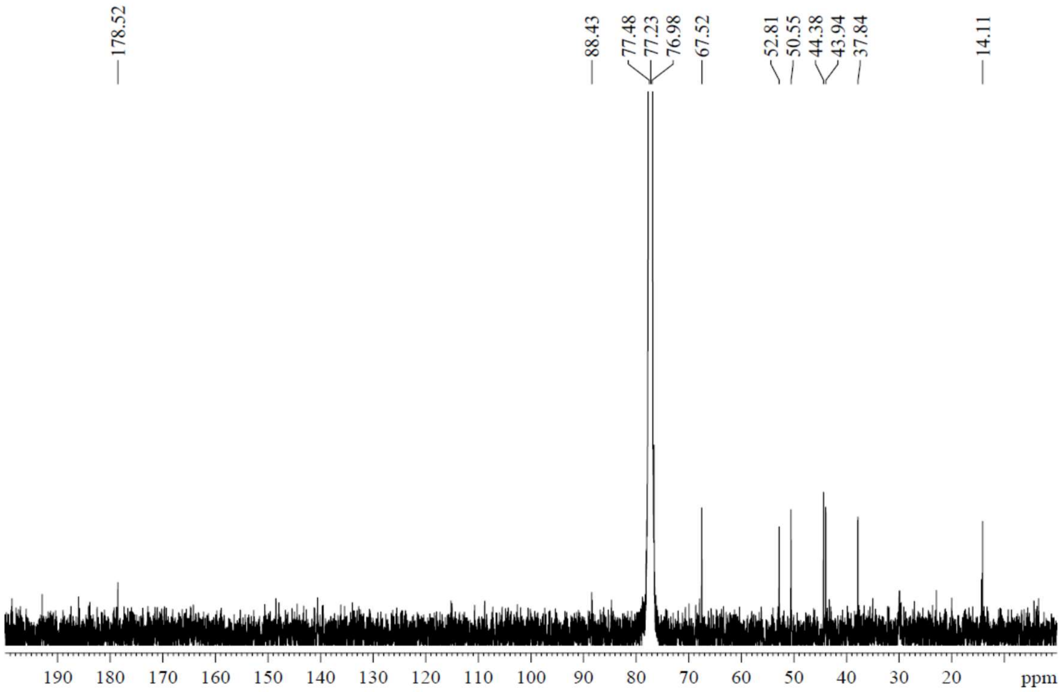

Fig. S3. ^{13}C-NMR spectrum of compound **1** ($CDCl_3$, 125 MHz).

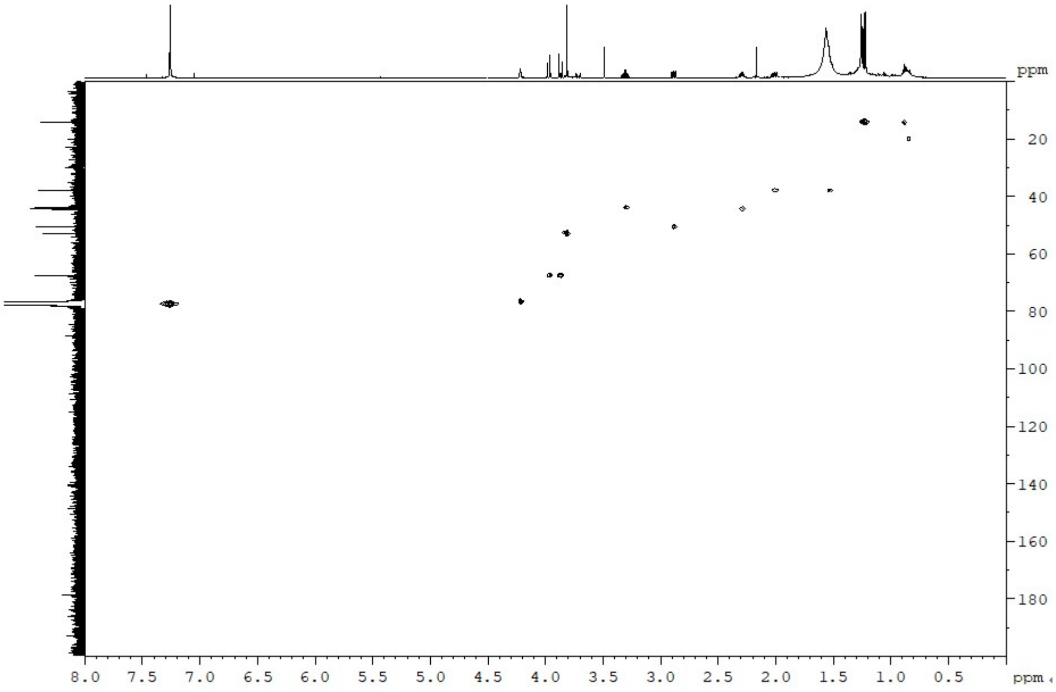

Fig. S4. HSQC spectrum of compound **1** ($CDCl_3$, 500 MHz).

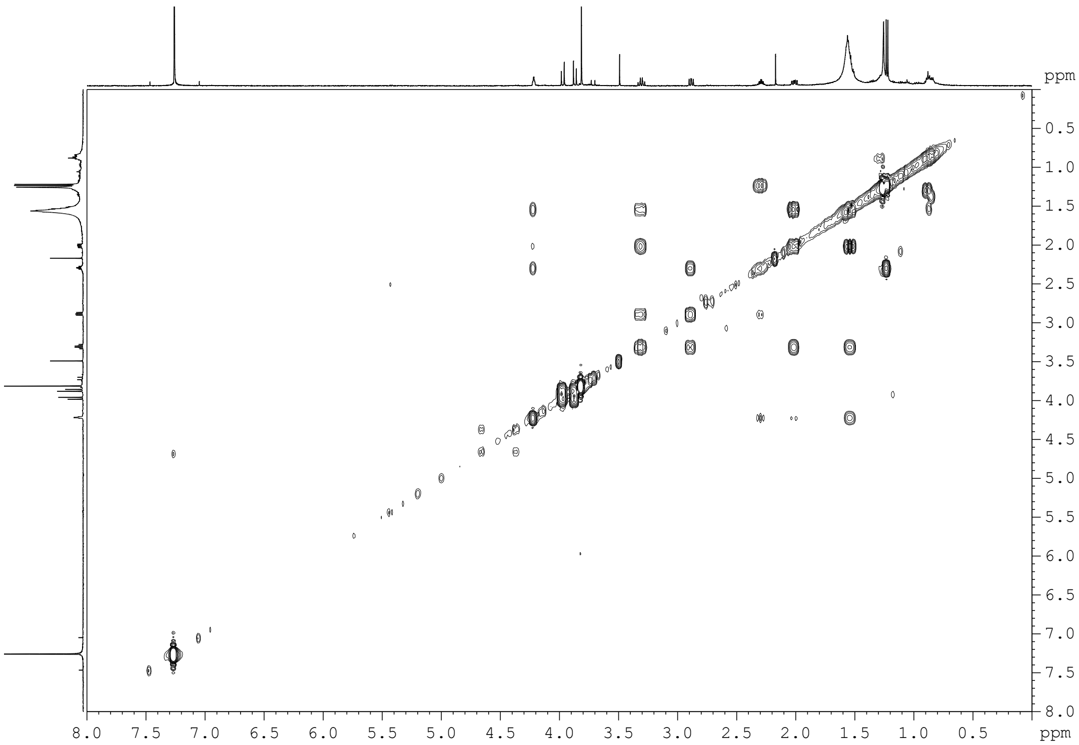

Fig. S5. ^1H-^1H COSY spectrum of compound **1** ($CDCl_3$, 500 MHz).

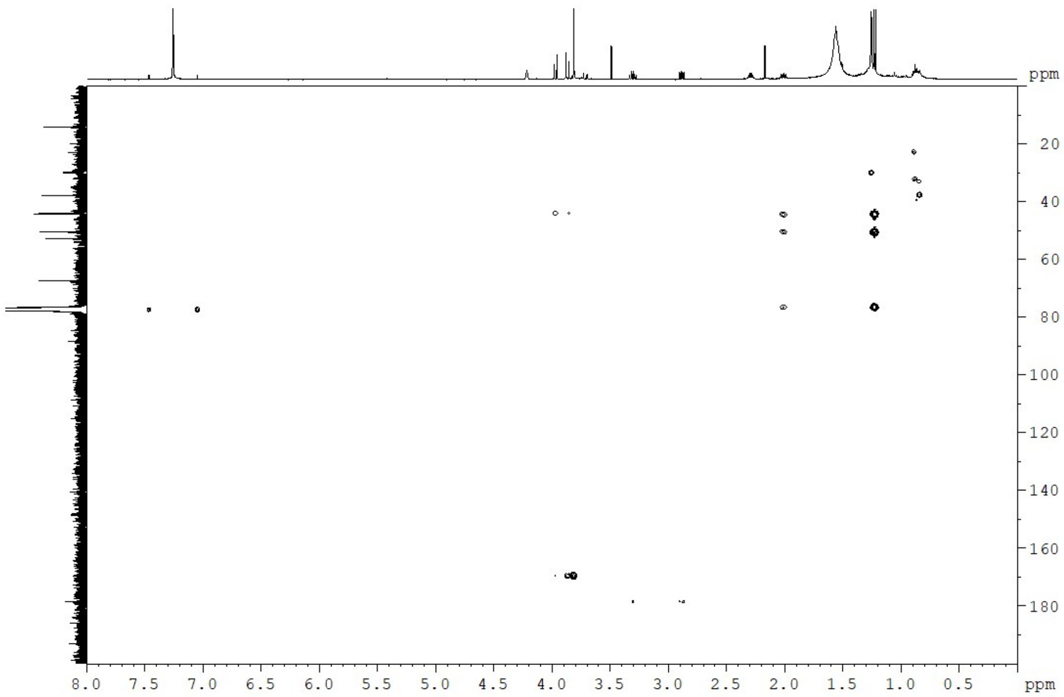

Fig. S6. HMBC spectrum of compound **1** ($CDCl_3$, 500 MHz).

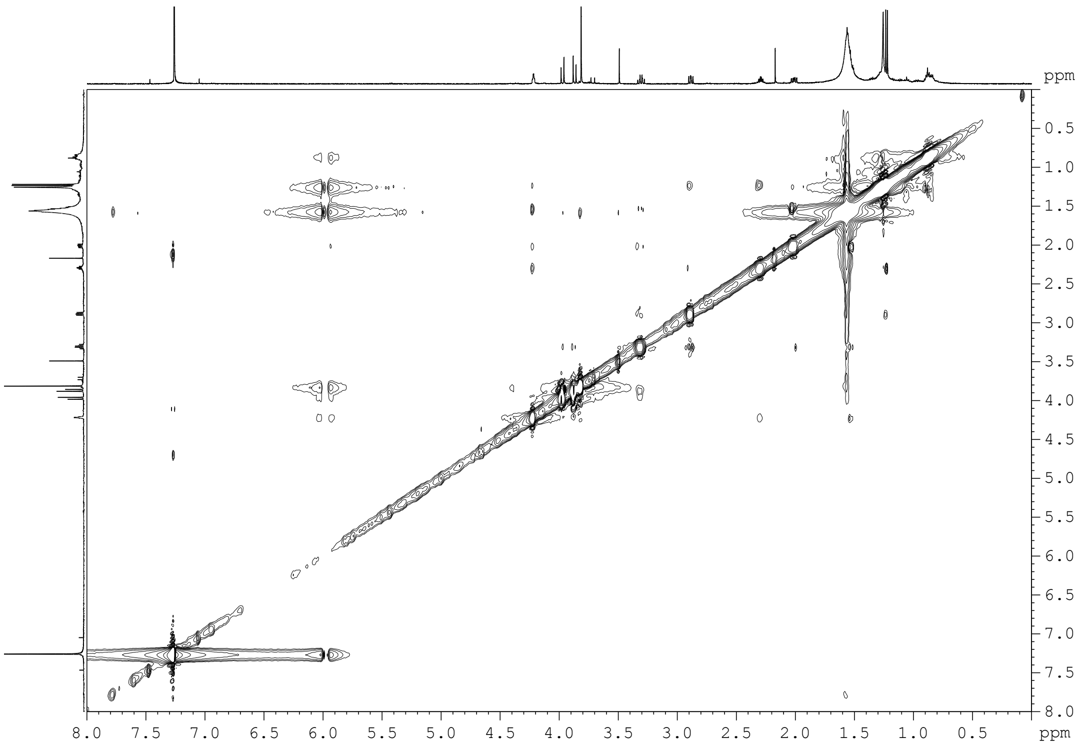

Fig. S7. NOESY spectrum of compound **1** ($CDCl_3$, 500 MHz).

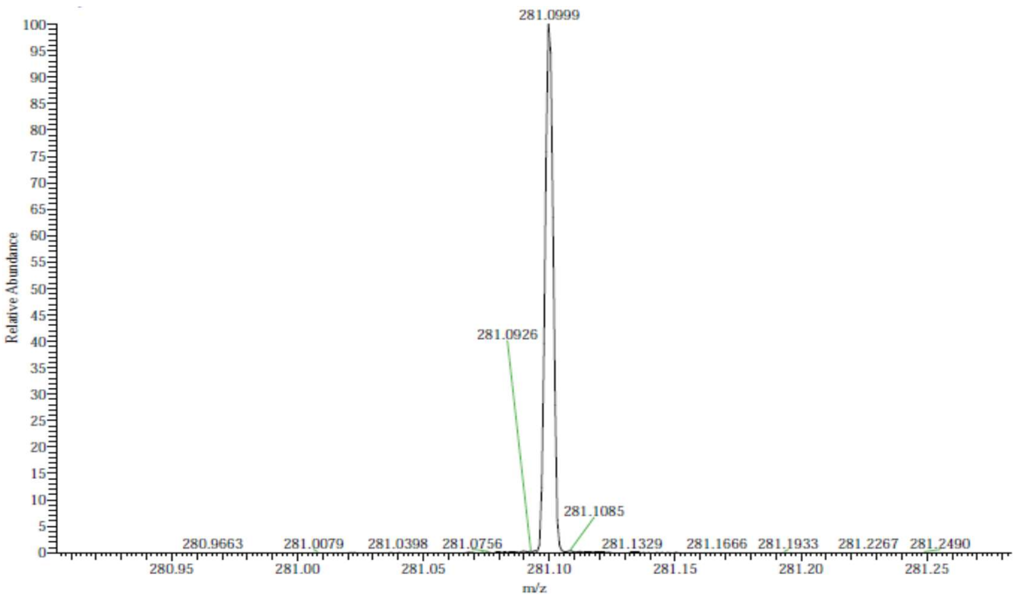

Fig. S8. HR-ESI-MS spectrum of compound **2.**

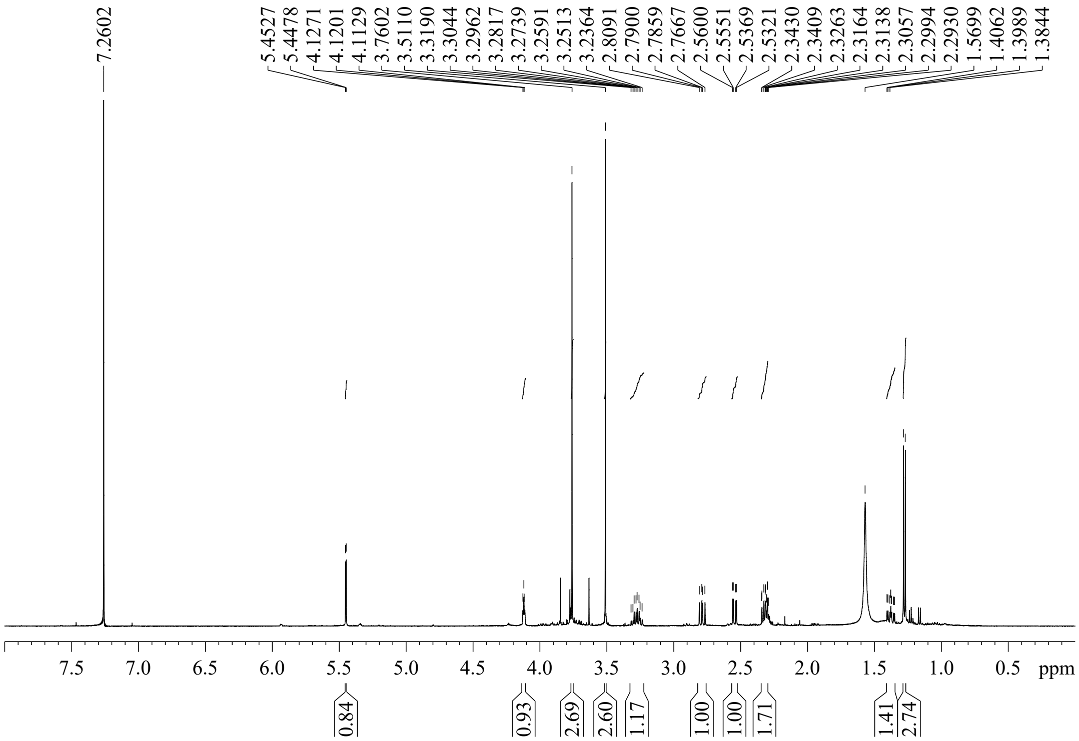

Fig. S9. ^{1}H-NMR spectrum of compound **2** ($CDCl_3$, 500 MHz).

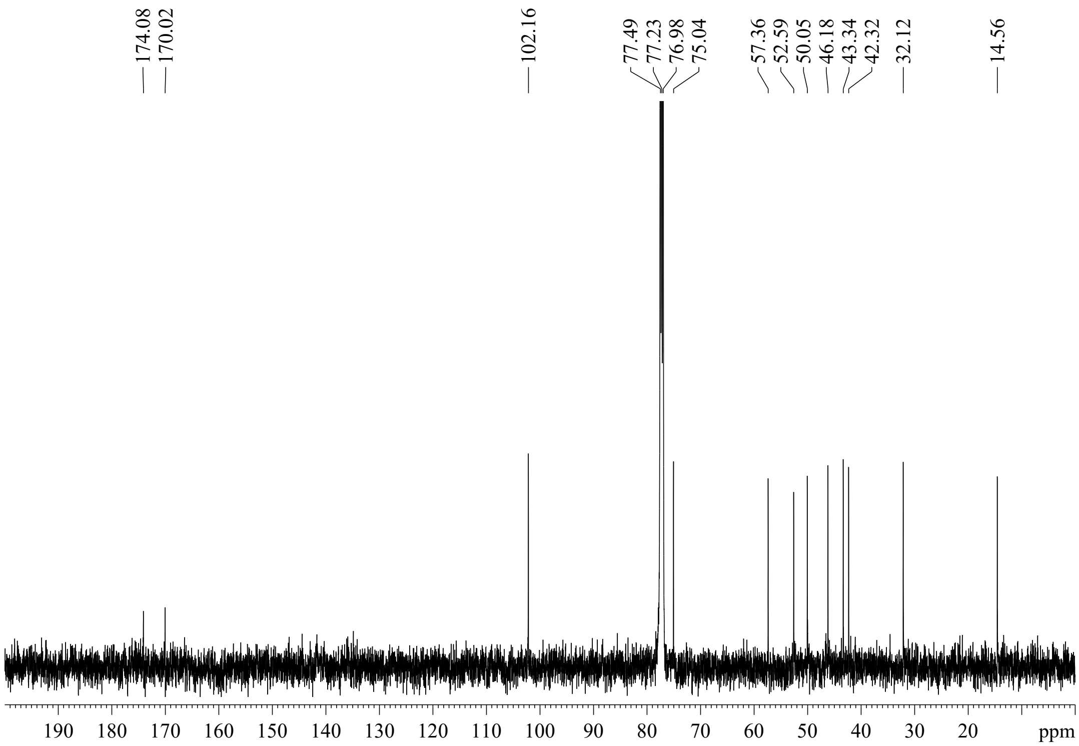

Fig. S10. ^{13}C-NMR spectrum of compound **2** ($CDCl_3$, 125 MHz).

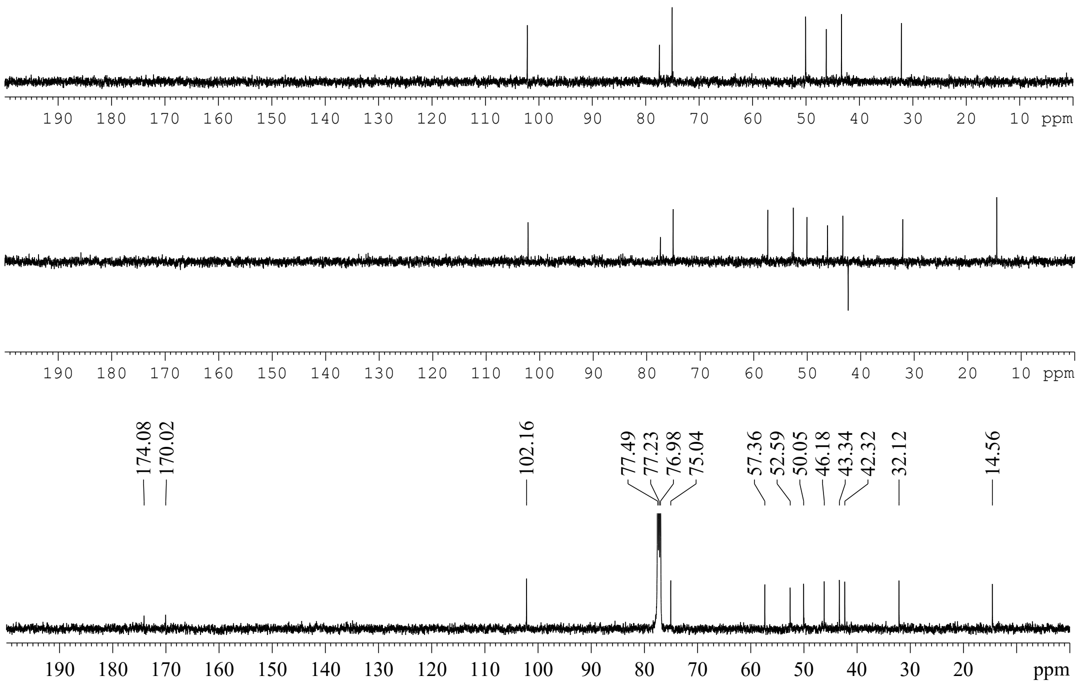

Fig. S11. DEPT spectrum of compound **2** ($CDCl_3$, 125 MHz).

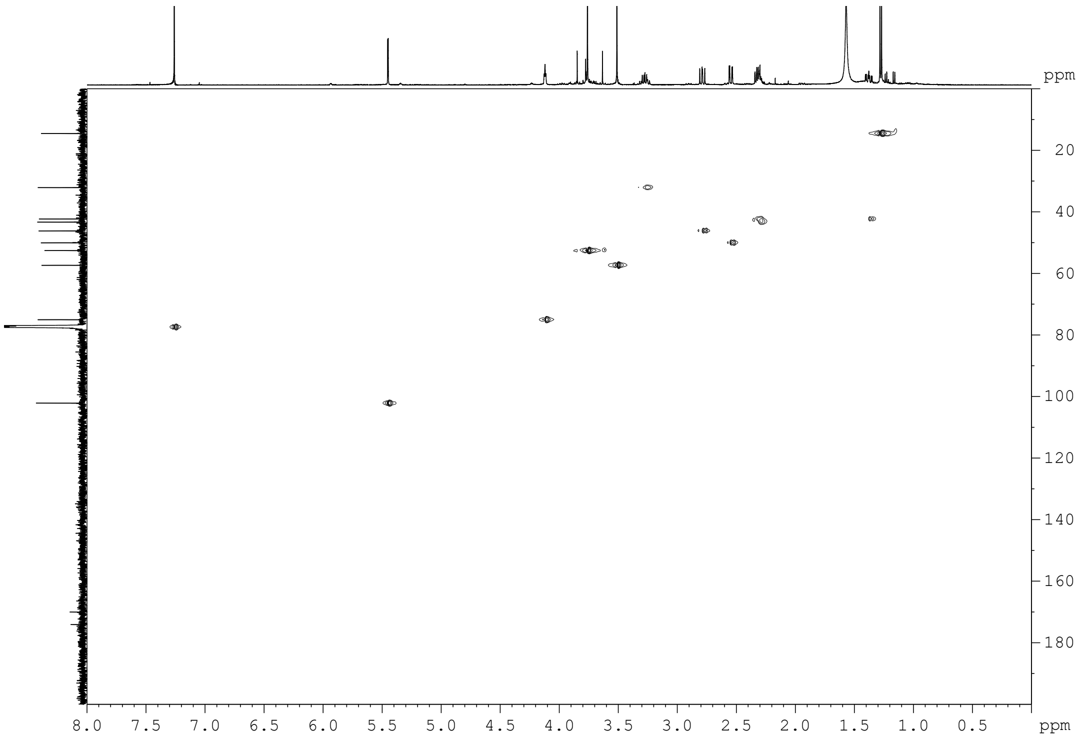

Fig. S12. HSQC spectrum of compound **2** ($CDCl_3$, 500 MHz).

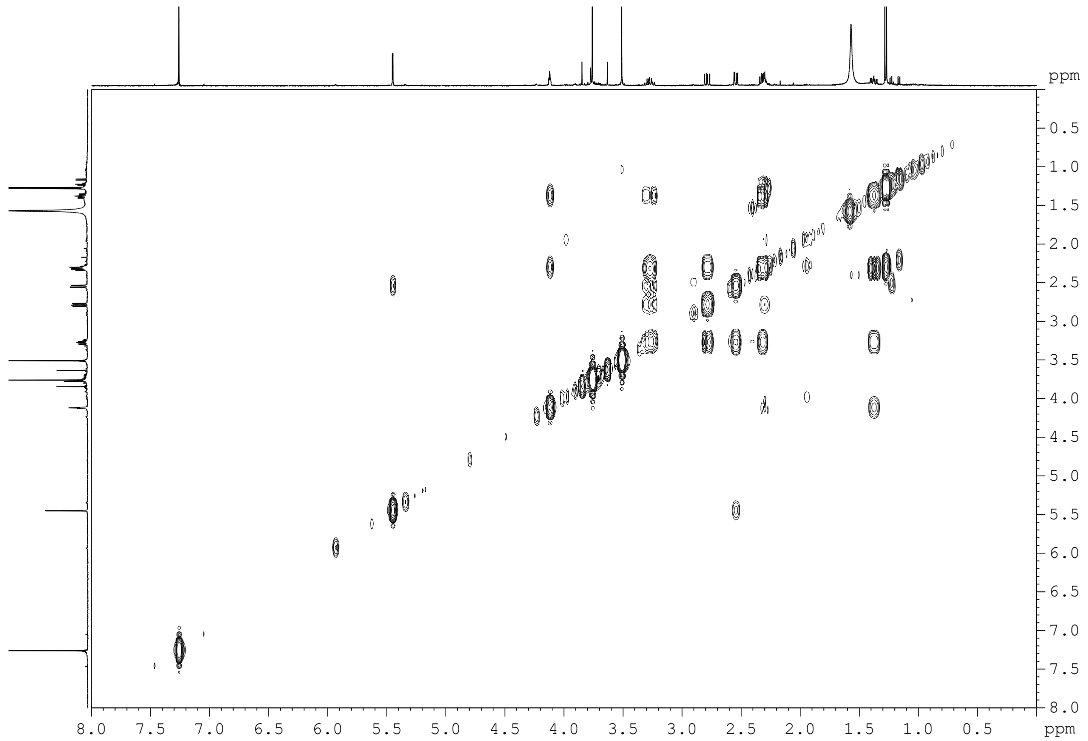

Fig. S13. ^{1}H-^{1}H COSY spectrum of compound **2** ($CDCl_3$, 500 MHz).

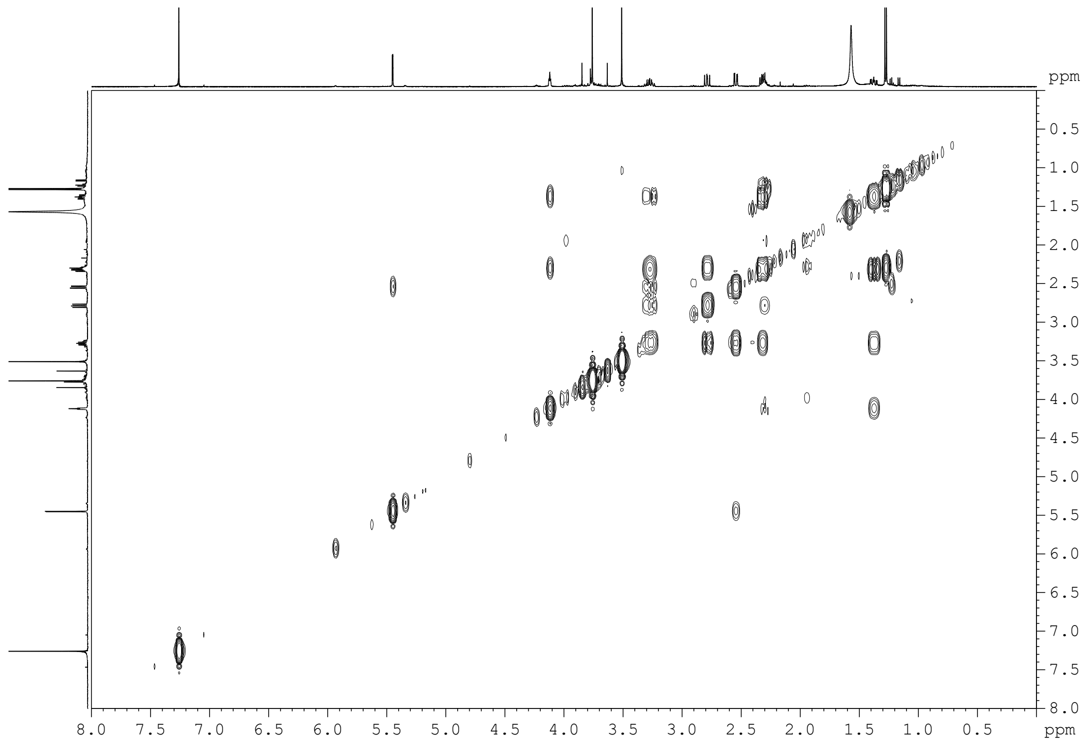

Fig. S14. HMBC spectrum of compound **2** ($CDCl_3$, 500 MHz).

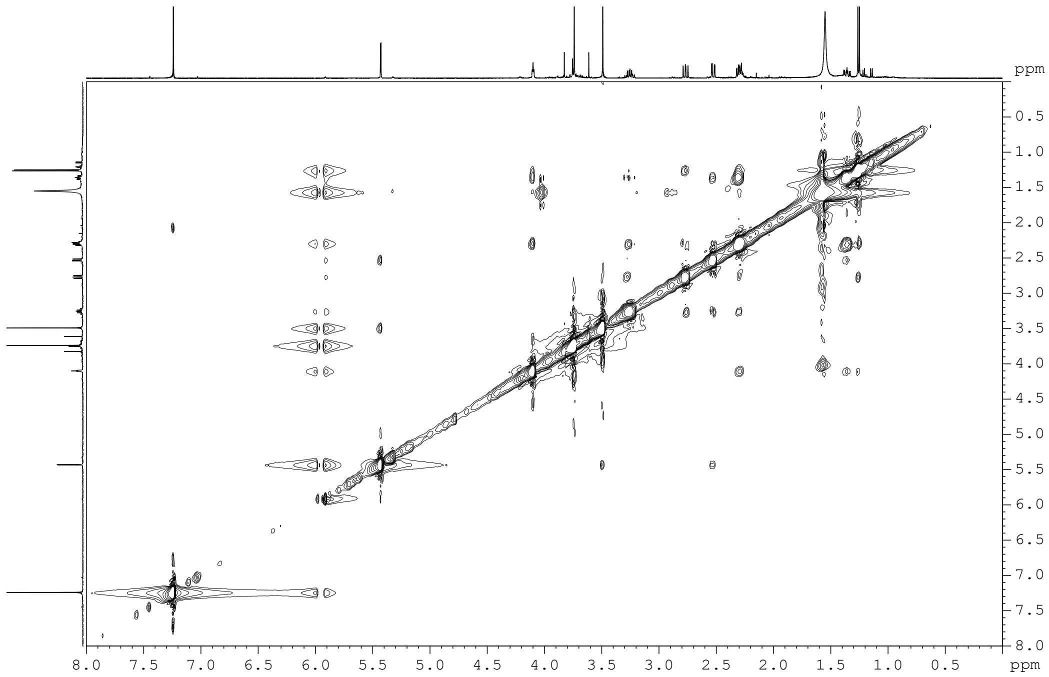

Fig. S15. NOESY spectrum of compound **2** ($CDCl_3$, 500 MHz).

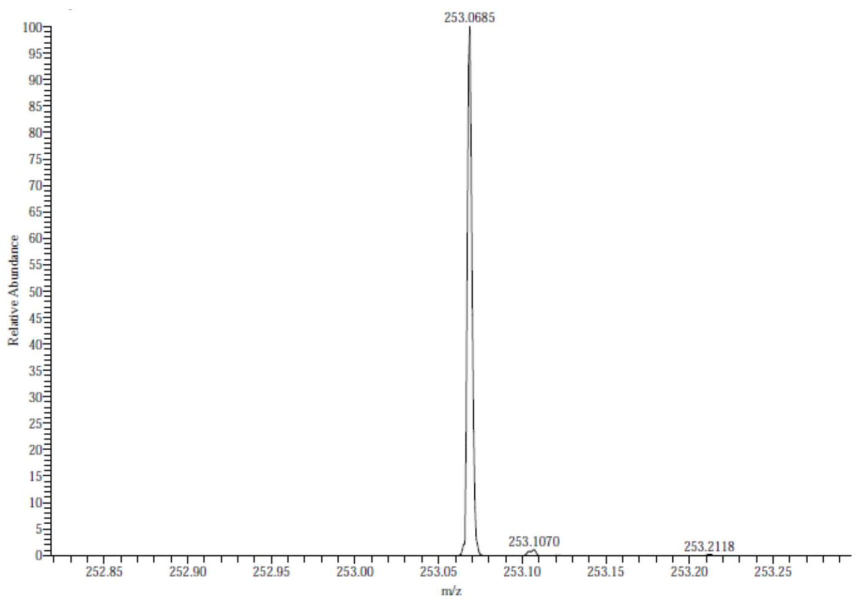

Fig. S16. HR-ESI-MS spectrum of compound **3**

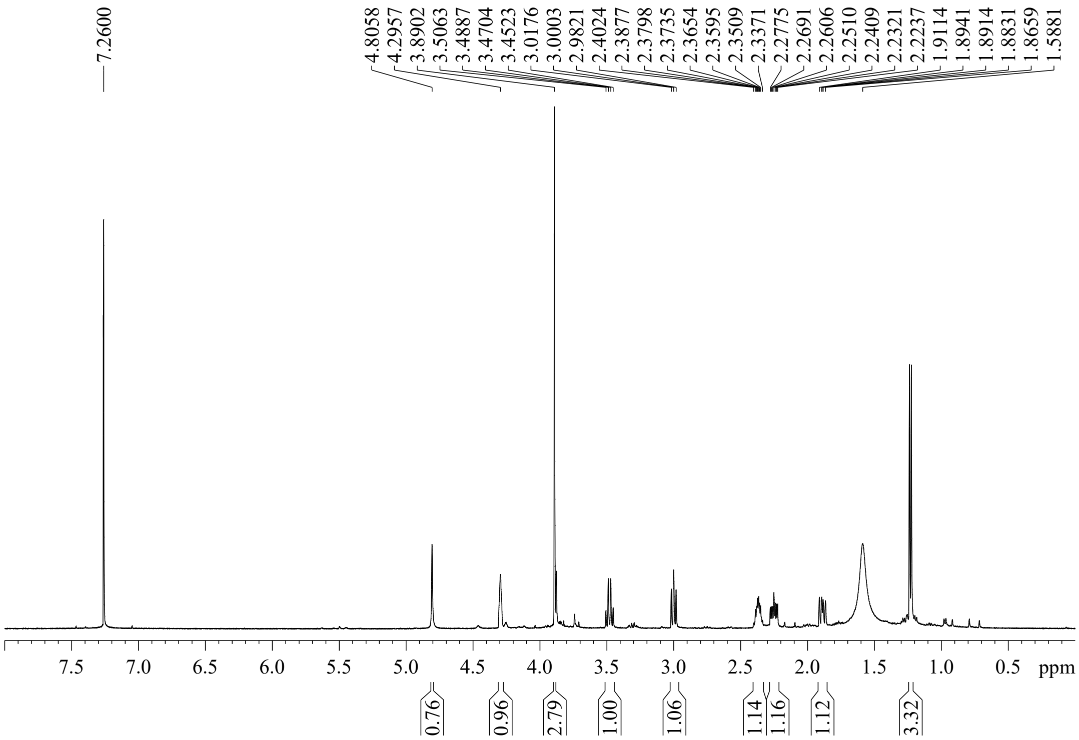

Fig. S17. ^{1}H-NMR spectrum of compound **3** ($CDCl_3$, 500 MHz).

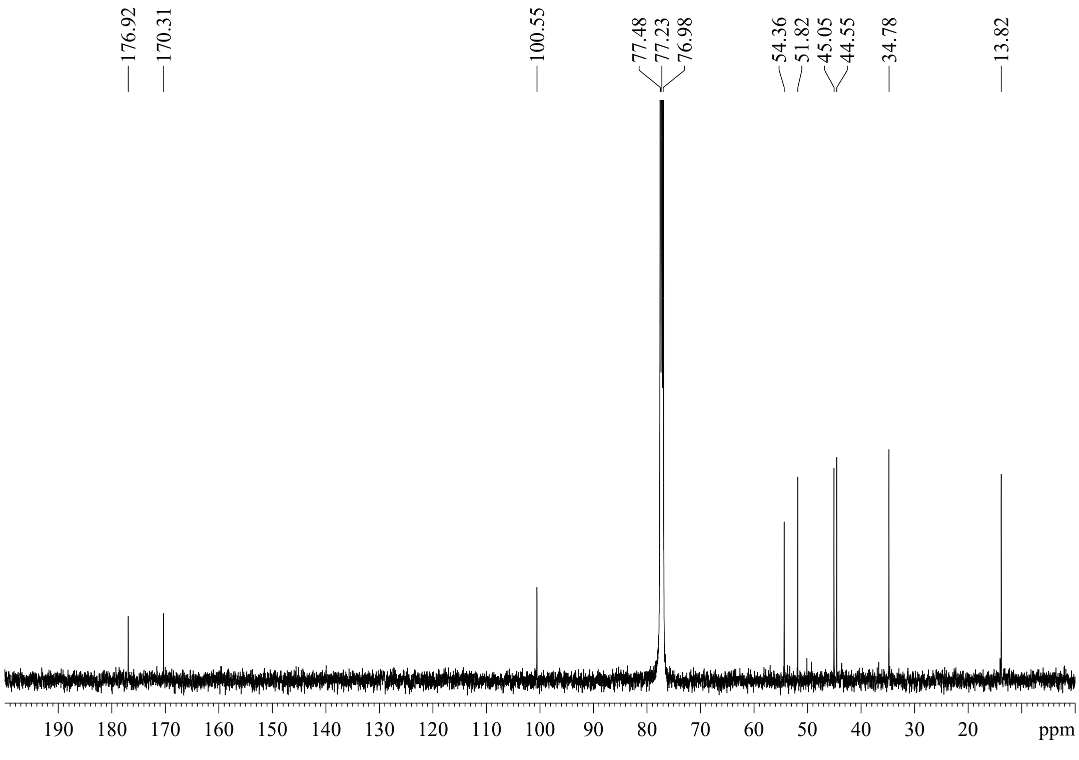

Fig. S18. ^{13}C-NMR spectrum of compound **3** ($CDCl_3$, 125 MHz).

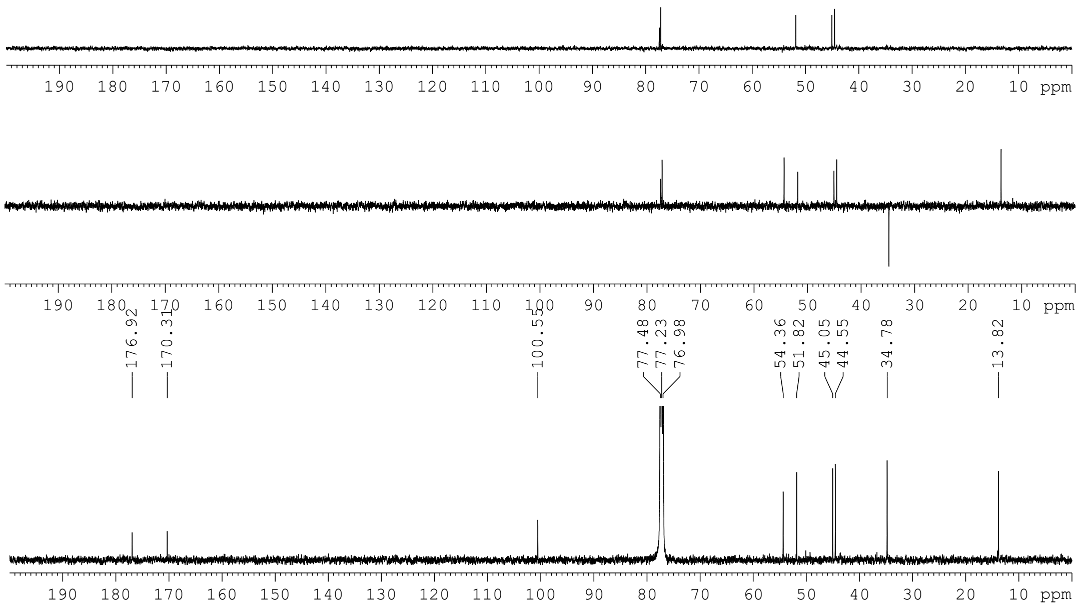

Fig. S19. DEPT-NMR spectrum of compound **3** ($CDCl_3$, 125 MHz).

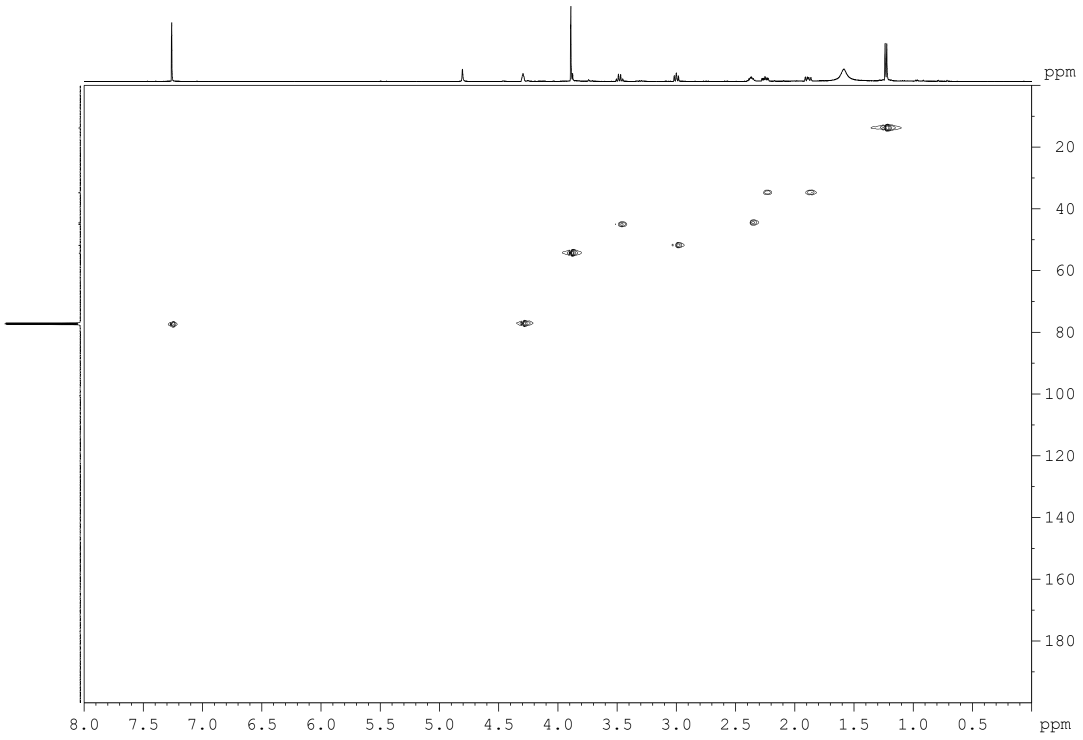

Fig. S20. HSQC-NMR spectrum of compound **3** ($CDCl_3$, 500 MHz).

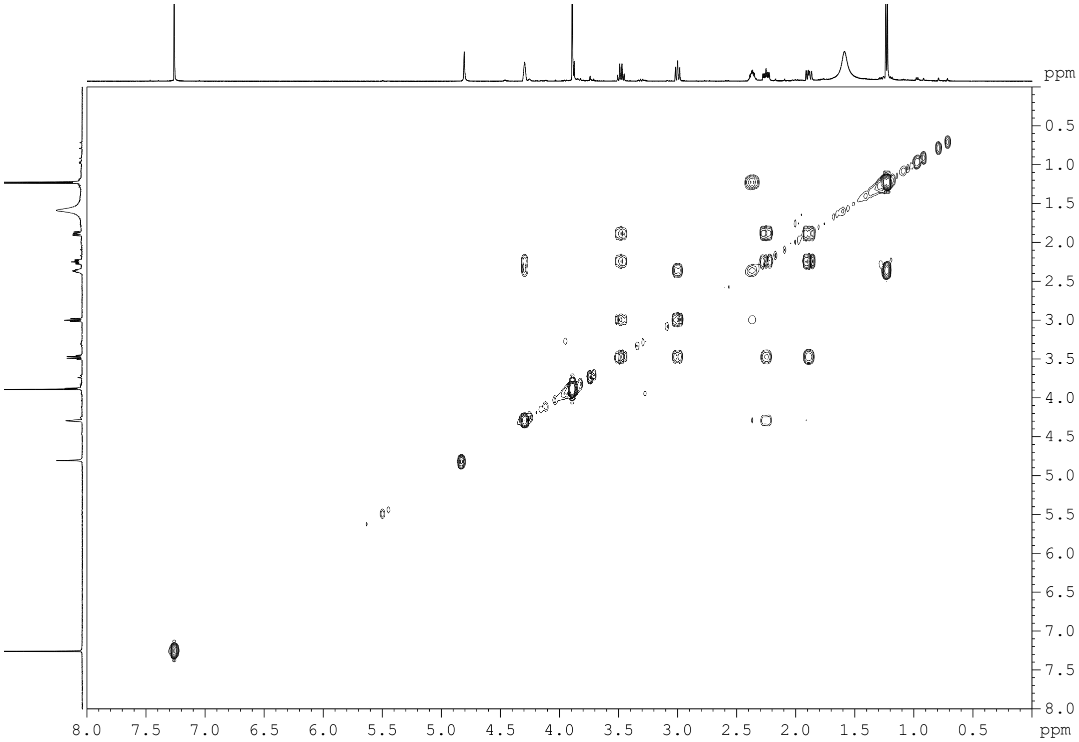

Fig. S21. ^1H-^1H COSY-NMR spectrum of compound **3** ($CDCl_3$, 500 MHz).

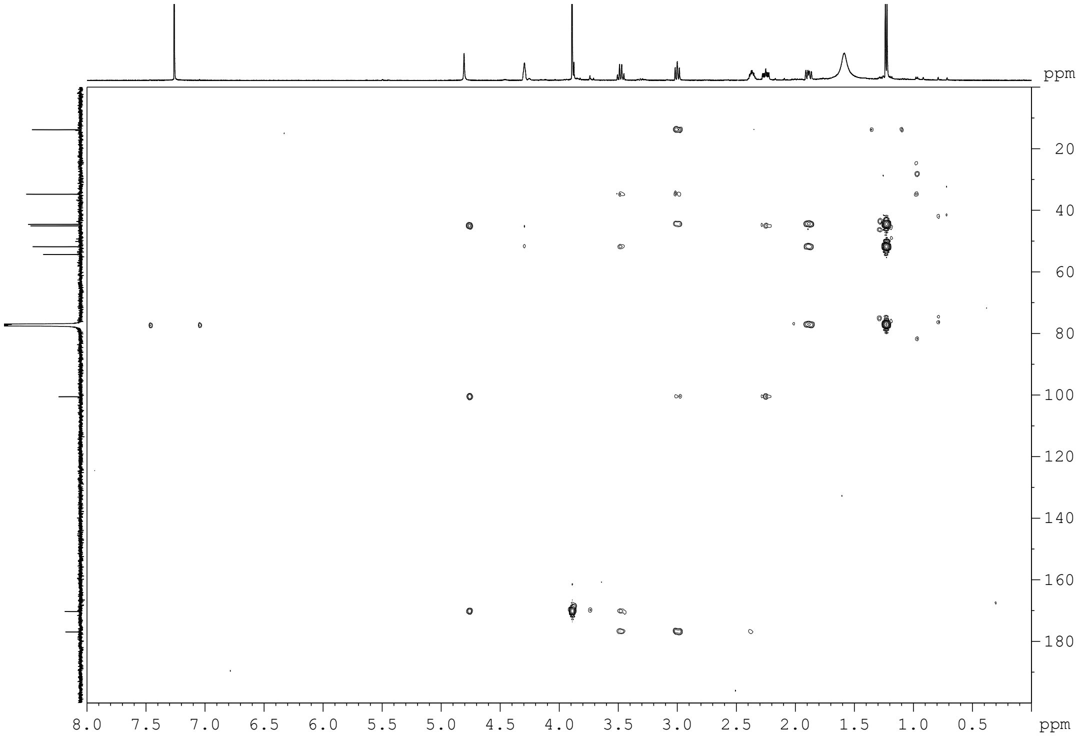

Fig. S22. HMBC-NMR spectrum of compound **3** ($CDCl_3$, 500 MHz).

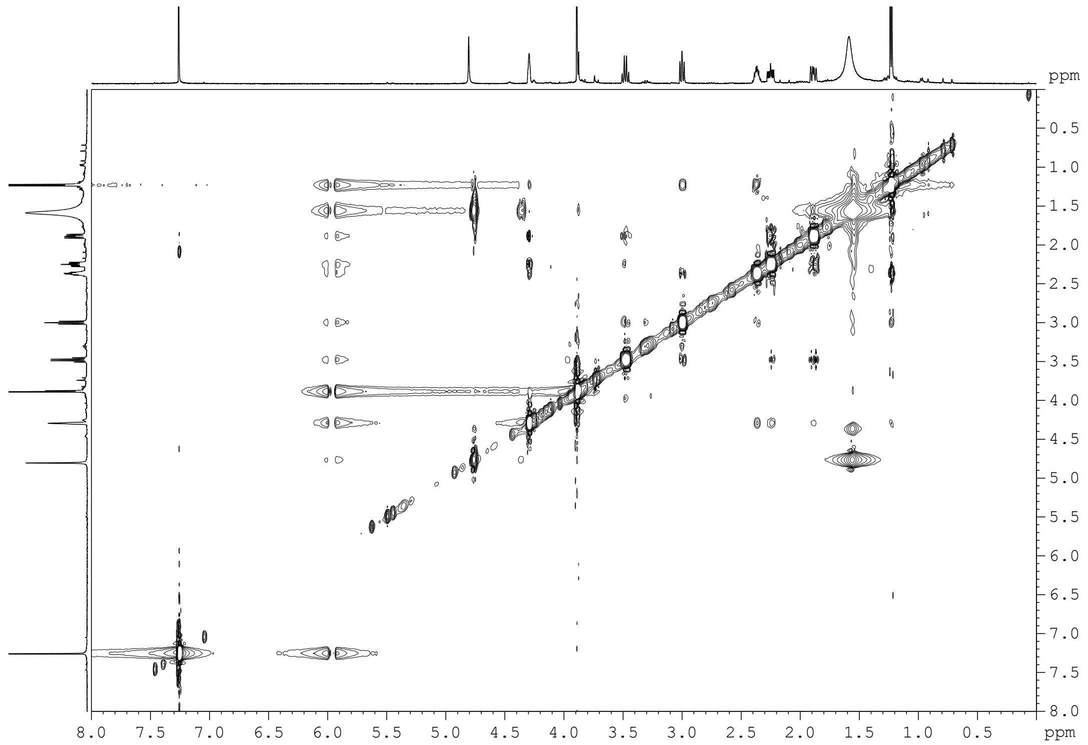

Fig. S23. NOESY-NMR spectrum of compound **3** ($CDCl_3$, 500 MHz)

Table S1. Cytotoxicity activity of extract, partition fractions and compounds **1–13** against Hep3B cell.

	**Dose** (**μg/mL**)	**Cell Viability** (**% of Control**)	**LD_{50}** (**μg/mL**)
Control		100	
MeOH extracts	62.5	93.90 ± 4.70	912.98 ± 3.95
	125	88.35 ± 7.27	
	250	83.99 ± 6.24	
	500	69.59 ± 3.86	
	1000	45.85 ± 3.95	
EtOAc fraction	62.5	86.04 ± 3.10	591.13 ± 4.99
	125	81.10 ± 5.78	
	250	73.30 ± 7.16	
	500	53.67 ± 9.49	
	1000	32.56 ± 4.99	
BuOH fraction	62.5	103.76 ± 3.38	>1000
	125	103.94 ± 5.58	
	250	103.53 ± 5.69	
	500	97.76 ± 10.46	
	1000	88.25 ± 6.49	
H_2O fraction	62.5	100.10 ± 1.57	>1000
	125	99.50 ± 3.38	
	250	99.50 ± 4.25	
	500	96.23 ± 2.92	
	1000	93.14 ± 2.72	
neonanin A (**1**)	6.25	104.07 ± 4.13	>1000
	12.5	105.29 ± 2.73	
	25	104.11 ± 3.30	
	50	103.86 ± 5.07	
	100	105.85 ± 3.58	
neonanin B (**2**)	6.25	100.63 ± 101.06	>100
	12.5	101.01 ± 102.61	
	25	103.18 ± 103.42	
	50	105.04 ± 101.72	
	100	106.69 ± 103.71	
neoretinin A (**3**)	6.25	101.40 ± 1.76	>100
	12.5	100.22 ± 1.40	

	25	99.34 ± 1.10	
	50	97.65 ± 2.52	
	100	95.42 ± 3.69	
6-hydroxy-7-methyl-1-oxo-4-carbomethoxyoctahydrocyclopenta[c]pyran (**4**)	6.25	102.15 ± 0.46	>100
	12.5	100.26 ± 2.39	
	25	99.52 ± 1.97	
	50	99.52 ± 1.66	
	100	96.46 ± 1.60	
4-epi-alyxialactone (**5**)	6.25	101.19 ± 1.79	>100
	12.5	103.08 ± 1.41	
	25	106.90 ± 3.35	
	50	105.63 ± 4.60	
	100	102.04 ± 1.73	
loganetin (**6**)	6.25	100.39 ± 0.70	>100
	12.5	99.88 ± 0.90	
	25	99.02 ± 1.23	
	50	98.64 ± 0.48	
	100	96.87 ± 1.67	
loganin (**7**)	6.25	97.67 ± 1.36	>100
	12.5	95.84 ± 4.76	
	25	95.88 ± 1.85	
	50	96.33 ± 2.77	
	100	92.76 ± 6.60	
phenylcoumaran - α′- aldehyde (**8**)	6.25	100.16 ± 5.42	>100
	12.5	101.20 ± 4.50	
	25	100.11 ± 3.38	
	50	97.54 ± 3.70	
	100	96.79 ± 8.63	
cleomiscosin A (**9**)	6.25	99.22 ± 1.42	>100
	12.5	102.69 ± 1.89	
	25	102.13 ± 1.39	
	50	104.83 ± 1.22	
	100	103.29 ± 0.72	
ficusal (**10**)	6.25	92.37 ± 0.56	85.36 ± 4.36
	12.5	84.77 ± 1.03	
	25	72.16 ± 0.59	
	50	48.56 ± 0.17	
	100	31.44 ± 1.61	

balanophonin (**11**)	6.25	101.88 ± 2.86	92.63 ± 1.41
	12.5	103.36 ± 0.67	
	25	101.68 ± 1.17	
	50	72.48 ± 3.41	
	100	47.78 ± 2.60	
vanillic acid (**12**)	6.25	99.33 ± 0.93	>100
	12.5	96.93 ± 2.71	
	25	95.93 ± 3.18	
	50	98.67 ± 0.47	
	100	94.18 ± 2.84	
p-coumaric acid (**13**)	6.25	91.48 ± 3.57	29.18 ± 3.48
	12.5	80.09 ± 5.08	
	25	64.14 ± 7.43	
	50	45.91 ± 5.92	
	100	26.65 ± 5.30	
Doxorubicin	0.039	8.30 ± 8.09	0.31± 0.08
	0.078	21.80 ± 3.12	
	0.156	40.72 ± 5.91	
	0.3125	53.44 ± 4.63	
	0.625	58.78 ± 1.22	
